# Right Ventricular Longitudinal Strain-Related Indices in Acute Pulmonary Embolism

**DOI:** 10.3390/medicina60101586

**Published:** 2024-09-27

**Authors:** Ioannis Tzourtzos, Lampros Lakkas, Christos S. Katsouras

**Affiliations:** 1 Second Department of Cardiology, University Hospital of Ioannina, 455 00 Ioannina, Greece; ioannistzourtz@gmail.com; 2 Department of Physiology, Faculty of Medicine, University of Ioannina, 451 10 Ioannina, Greece; ftpcavalier52@gmail.com; 3 First Department of Cardiology, University Hospital of Ioannina, 455 00 Ioannina, Greece; 4 Faculty of Medicine, School of Health Sciences, University of Ioannina, 451 10 Ioannina, Greece

**Keywords:** right ventricular strain, speckle-tracking echocardiography, pulmonary embolism

## Abstract

Pulmonary embolism (PE) is correlated with serious morbidity and mortality. Efforts have been made to establish and validate mortality predictive scores based mainly on clinical parameters. Patients with PE and traditional indices of echocardiographic right ventricular (RV) dysfunction or pressure overload have a higher probability of a worse outcome. During the last two decades, studies regarding the use of two-dimensional speckle-tracking echocardiography (2DSTE) and its derived indices in the setting of acute PE have been conducted. In this comprehensive review of the literature, we aimed to summarize these studies. Safe conclusions and comparisons among the reviewed studies are prone to statistical errors, mainly because the studies published were heterogenous in design, different 2DSTE-derived parameters were tested, and different clinical outcomes were used as endpoints. Nonetheless, RV strain indices and, more commonly, regional longitudinal strain of the RV free wall have shown a promising correlation with mortality, assisting in the differential diagnosis between PE and other acute or chronic disorders.

## 1. Introduction

Venous thromboembolism (VTE) is a leading cause of both cardiovascular-disease-attributed morbidity and mortality. The overall incidence of VTE is 0.75 to 2.69 per 1000 individuals per year in the Western world [1]. The incidence ranges from being rare in children to relatively common in the elderly (2.91–12.04/1000/year among those aged ≥ 80 years), while it is many times higher in in-patients compared to the general population and lower in studies conducted in Asian countries [1,2,3]. One-third to one-half of the VTE cases are attributed to pulmonary embolism (PE), and these patients have higher short- and long-term mortality compared to patients with deep vein thrombosis (DVT) [1,4]. The first 30 days after the diagnosis of PE is the higher death risk period (31% in PE, 3% in DVT, and 0.4% in individuals from the general population matched by age and sex) [4].

In patients with suspected PE, echocardiography can be used for diagnostic reasons since this syndrome is associated with right ventricular (RV) dysfunction (_d_) or pressure overload (_PO_). In patients with suspected high-risk PE, echocardiography is useful to detect signs of RV_d/PO_. The absence of these indices almost excludes PE [5]. In the latest European guidelines for the diagnosis and management of acute PE, the role of echocardiography in non-high-risk PE has been upgraded: beyond its prognostic value, it is stated that it may be useful in differential diagnosis of patients with acute dyspnea [5]. Furthermore, it is reasonable to perform a TTE in every patient with suspected or diagnosed PE in terms of having qualitative and quantitative baseline measurements to make a comparison with follow-ups, since due to the peculiar anatomy of RV, there is no single echocardiographic index providing accurate details on RV geometry and function.

Over the years, “traditional” echocardiography-derived indices have been used to serve the aforementioned needs. Newer techniques, such as two-dimensional speckle-tracking echocardiography (2DSTE), have been developed in the last decades and, while they have been tested in a variety of conditions, until today they have not earned a spot in PE. In this review, we aimed to summarize the currently available published data regarding the 2DSTE-derived indices, specifically for the right ventricle in the setting of acute pulmonary embolism.

## 2. Echocardiogram in PE

### 2.1. Echocardiogram in Patients with High-Risk PE

Patients with hemodynamic instability, defined as cardiac arrest, cardiogenic shock, or persistent hypotension, have a higher risk of death during the first 30 days after the diagnosis of PE (high-risk PE) [5]. Most medical societies justify thrombolysis in patients with suspected high-risk PE if echocardiographic signs of RV_d/PO_ are detected, as well as right heart thrombi. However, when the patient is stabilized, they recommend confirmation of PE by computed tomography pulmonary angiography (CTPA). Only the British Thoracic Society guidelines recommend bedside transthoracic echocardiography (TTE), transesophageal echocardiography, or lower limb vein compression ultrasound in patients with suspected PE and hemodynamic instability to adopt PE as the final diagnosis if RVd, right heart thrombi, or DVT is detected [5,6,7]. Meanwhile, other medical societies (e.g., the American Society of Hematology and American College of Physicians) do not provide any recommendation on the usage of TTE as a guidance to fibrinolysis in patients not already diagnosed with PE [7,8]. The European Society of Cardiology/European Respiratory Society (ESC/ERS) guidelines state that in suspected high-risk PE, the absence of echocardiographic signs of RV_d/PO_ “practically” excludes PE, and specific echocardiographic findings (60/60 sign, McConnell sign, and right heart thrombi) justify thrombolysis if immediate CTPA is not feasible [5,6,7]. Moreover, the method excludes other causes of shock, such as pericardial tamponade, aortic dissection, or acute valvular dysfunction.

### 2.2. Echocardiogram in Non-High-Risk PE

According to guidelines from medical societies/organizations, TTE is not mandatory during diagnostic work-up in non-high-risk PE (hemodynamically stable patients), mainly due to unsatisfactory sensitivity and a negative likelihood ratio [7,8,9]. However, echocardiographic signs of RV_d/PO_ are valid for patient classification as intermediate-risk PE, even if all other risk factors are absent. Based on the results of a validate clinical score (pulmonary embolism severity index (PESI)), echocardiogram (RV_d/PO_), CTPA (the ratio of RV/left ventricular (RV/LV) diameter), and the presence of markers of myocardial injury (troponin), non-high-risk PE can be classified as either low- or intermediate-risk (which is further stratified as intermediate–high or intermediate–low risk) [5]. Besides an RV/LV ratio > 0.9, as proposed by the American Heart Association, and ≥1, as proposed by ESC, the following markers indicating RV_d/PO_ in TTE have been shown to have an association with an unfavorable prognosis: large RV diameter (>42 mm at the base or >35 mm at the mid-level), McConnell sign, D-shape in parasternal short axis view (PSAX), distended inferior vena cava (IVC) with diminished inspiratory collapsibility, 60/60 sign, tricuspid annular plane systolic excursion (TAPSE) < 16 mm, and S′ < 9.5 cm/s. Of these, the RV/LV ratio and TAPSE are most frequently reported [5], but a meta-analysis of 22 studies showed that TTE has low sensitivity and a negative predictive value and, therefore, no TTE-related indices can rule out PE [10]. On the contrary, TTE has higher specificity than sensitivity, namely, the presence of thrombus in the right heart (specificity 100% and sensitivity 4%) or the McConnell sign (specificity > 95% and sensitivity 16–50%) [11]. Unfortunately, the true numbers regarding the sensitivity of TTE in PE may be different and possibly lower than that reported in studies and meta-analyses. In most of the studies that examined TTE in PE, only a few patients with PE underwent TTE, and there was no detailed information regarding the initial group of potentially included patients [12]. In a recently published study of unselected patients with documented PE, the indices (in a group) had a sensitivity of about 20% [13]. Regarding the risk of 30-day mortality after PE, data from the ICOPER Registry showed that TTE had a low positive predictive value (<20%), but the main problem was the relatively low negative predictive value (about 90%). If we consider the predicted mortality of PE, then it is easily explained why the negative likelihood ratio of the method (0.50–0.90) is not satisfactory (only very low numbers, <0.2, afford us a useful discriminating ability) [14,15]. Unfortunately, CTPA and the ratio of the RV/LV diameter face the same problem [16]. To improve the diagnostic accuracy and prognostic role of TTE in patients with PE, non-conventional indices have also been tested. However, in clinical practice, in our opinion, every patient with suspected or diagnosed PE has to undergo a TTE: before the diagnosis, since it may be useful in the differential diagnosis of other causes of acute dyspnea or chest pain, and at the time or after the diagnosis of PE for prognostic reasons and comparisons with future TTEs.

### 2.3. Speckle-Tracking Echocardiography: A Sensitive Index of RV Systolic Function

How can we explain that in most non-high-risk patients, the overload of RV after PE does not have an effect on RV function based on the previously mentioned TTE indices? The complex anatomy, the thinner RV wall, and the peculiar geometry and physiology of RV may be responsible for this, and it is possible that these factors restrict RV_d/PO_ to be detected in TTE [17]. A more sensitive method to subtle changes in RV function could add to the diagnostic accuracy of TTE in PE.

**Figure 1:**
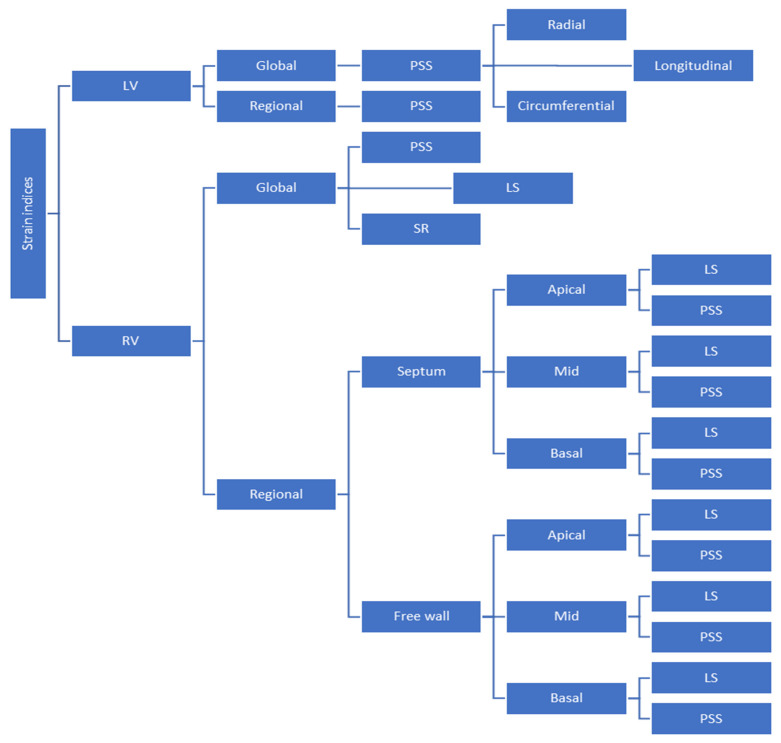
Strain-related indices used in the reviewed clinical studies. Abbreviations: LS = longitudinal strain, LV = left ventricle, PSS = peak systolic strain, RV = right ventricle, and SR = strain rate.

The RV mechanical strain and strain rate can be measured using echocardiography and magnetic resonance imaging. Obviously, the former is more easily and commonly used in everyday clinical practice. The most common RV strain index is regional or global RV longitudinal strain (RVLS), which has been shown to be feasible and accurate [18]. RVLS by 2DSTE is a very sensitive index of RV systolic performance and has been used for disease assessment and as a prognostic marker in patients with pulmonary hypertension, heart failure, congenital heart disease, cardiomyopathies, and valvular heart diseases [18]. We must bear in mind that the deep muscle fibers of the dominant inner layer of the RV are longitudinally aligned from base to apex, and longitudinal shortening accounts for 75–80% of RV contraction [19]. During the last two decades, small clinical studies have examined the role of RVLS in the clinical setting of PE, while guidelines do not recommend anything about it. These studies were heterogeneous in design, prospective or retrospective, were conducted in different centers all around the world, and a small number of selected or non-selected and consecutive or non-consecutive patients were included. The definitions of “massive” or “sub-massive” PE differed among the studies, and data regarding the time from PE diagnosis to 2DSTE were not always thoroughly provided. Furthermore, the exclusion criteria also differed (e.g., patients with heart failure or chronic obstructive pulmonary disease were excluded in some of the studies). Finally, not all the investigators used the same 2DSTE parameter (e.g., global RVLS, free-wall RVLS, or just mid-free-wall RVLS), or they even used different cut-off values, which did not allow comparisons to be made (Figure 1).

No study investigating RVLS enrolled exclusively patients with high-risk PE, since some studies included both non-high-risk and high-risk PE patients. However, not even traditional indices have been tested in high-risk PE. On a theoretical basis, RVLS does not have any clinical value in patients in cardiogenic shock above the already impacted “traditional” indices of RV_d/PO_ in TTE, and any delay in reperfusion therapy for these patients to measure RVLS could be catastrophic. Whether RVLS has any clinical meaning in patients treated with interventional therapies is also unknown [20].

## 3. Clinical Studies with 2DSTE in PE (Table 1)

### 3.1. The First Published Observation and the First Published Study Concerning Strain Analysis in Patients with PE

Twenty years ago, in 2004, Kjacrgaard et al. published their observations about RVLS in three patients with PE and RV dilatation without shock at presentation. They noticed that the strain pattern of the middle part of the RV free wall was stretched in systole, while the contractile function was preserved in the basal and apical segments. They mentioned that this was in concordance with McConnell’s sign. Two months later, the strain pattern was normalized. Moreover, they stated that Doppler tissue imaging of the RV is easily measured, having a satisfactory repeatability [21].

Then, 3 years later, in 2007, Park et al. published the results of a small study regarding the use of RVLS in the acute setting of PE in 24 (14 female, aged 69 ± 10 years) patients. Ten patients received thrombolysis (intravenous tissue plasminogen activator, 100 mg for 2 h), four had a contraindication to thrombolysis, and four refused it, while only six had no cardiogenic shock on admission. Baseline 2DSTE was performed on the day of admission, and it was repeated 4–34 days later.

**Table 1:** Summary of the reviewed clinical studies (listed from older to the most recently published).

First Author (Published Year) [Ref.]	Type of Study	Clinical Scenario	Type of Strain Used	Number of Patients in Each Group/Arm PE (+/− Other) Control		Main Results (Regarding PE-Related Findings)
Kjaergaard et al. (2004) [21]	Prospective, consecutive patients	Sub-massive PE before and after thrombolysis and at 2 months follow-up	RVLS of the RV free wall	3	−	Middle part of RV-FW had low or positive strain that normalized after thrombolysis (apical and basal segments had unaffected strain)
Park et al. (2007) [22]	Prospective, non-consecutive patients	PE before and after treatment (anticoagulation +/− thrombolysis)	PSS of the RV free wall	24	−	PSS of middle and apex part of RV-FW were impaired at the baseline and both improved after treatment (changes were more pronounced in the middle part)
Sugiura et al. (2009) [23]	Prospective, non-consecutive patients	Massive or sub-massive PE before and after primary treatment ^Ɨ^ vs. control group (age- and sex-matched healthy subjects)	Global and regional RV PSS	23	23	PSS was reduced in all segments except for the basal and middle septum (accordingly, global RV PSS was also reduced), in comparison to the control group * After treatment PSS improved in all initially affected segments except for the basal RV lateral wall, which in conjunction with the still impaired middle RV lateral and basal septum segments led to a reduced global RV PSS (however, it improved compared to the global RV PSS before treatment administration) *
Park et al. (2010) [24]	Retrospective, non-consecutive	Acute CP due to massive PE vs. patients with chronic CP due to severe COPD	Regional RV PSS	24 (28 COPD)	-	PSS of the middle segment of RV-FW was diminished in patients with acute CP compared to those with chronic CP due to severe COPD * Values of PSS of the middle segment of the RV free wall > −12.2% could predict acute CP (sensitivity 83.3%, specificity: 78.6%)
Takamura et al. (2011) [25]	Prospective, consecutive patients	Massive or sub-massive PE before and after primary treatment ^Ɨ^ vs. control group (age- and sex-matched healthy subjects)	Global and regional LV PSS in the three contractile directions	25	25	Global and regional radial PSS were reduced in patients with PE on admission and did not recover to normal values after treatment compared to the control group * Global and regional longitudinal PSS, apart from the basal lateral wall, were reduced in the setting of PE. After treatment: PSS in the basal and middle inferoseptum were still reduced, while PSS improvement of the apical segments and middle lateral wall led to a global PSS comparable to that of the control group * Global and regional circumferential PSS were diminished upon admission and restored to values comparable to those of the control group after treatment *
Platz et al. (2012) [26]	Retrospective, non-consecutive patients	PE vs. control group (age- and sex-matched subjects with no evidence of structural or valvular heart disease on echocardiography)	Global and regional RVLS	75	30	Global RV-FW and septal strain was affected in the PE subjects compared to the control group Except for the apical septum, regional RVLS was significantly reduced in all the other RV segments * All three segments of the RV-FW had significantly affected strain rates * Global RVLS did not differ between patients with and without McConnell sign
Ichikawa et al. (2013) [27]	Retrospective, non-consecutive patients	PE or CPAH vs. control group (age-matched normal subjects)	Global and regional RV and LV PSS	37 (36 CPAH)	33	Both groups showed a significant reduction in global RV longitudinal PSS compared to the control group The reduction in the PSS of the basal and middle part of the RV-FW was greater in PE * In the PE group, global PSS of the LV was reduced in comparison to the other 2 groups in almost all directions (longitudinal, radial, and circumferential), with only the PSS of the basal parts of the longitudinal axis being unaffected compared to the control group In the CPAH group, global PSS was maintained only in the longitudinal axis due to the intact PSS in the apical and lateral LV segments compared to the control group *
Vitarelli et al. (2014) [28]	Prospective, non-consecutive patients	Intermediate-risk PE at the onset of the acute episode and after median follow-up periods of 1 and 6 months vs. control group (sex- and age-matched healthy subjects)	RVGLS and RVFWS	66	66	Patients diagnosed with PE had lower RVGLS and lower RVLS in all RV-FW segments, with the mid-part being the most affected * Among 3D-RVEF, RVSP, and mid-FW wall RVLS, the latter showed a statistically significant reversal at 1-month follow-up, earlier than the others * Besides 3D-RVEF, mid-free-wall RVLS showed a statistically significant correlation with 6-month adverse outcomes ^¥^ Mid-FW RVLS using a cut-off value > −12% could predict adverse outcomes ^¥^ at 6 months (sensitivity, 84%; specificity, 74%) *
Khemasuwan et al. (2015) [29]	Retrospective, non-consecutive patients	Patients with PE admitted to the ICU at some point during their hospitalization	Global and regional RVLS	110 out of a total of 211	-	Global and regional RVLS were not associated with short-term (during hospitalization) or long-term mortality but were associated with the need of mechanical ventilation during the ICU stay
Wright et al. (2016) [30]	Retrospective, non-consecutive patients	Non-high-risk PE vs. control group (divided into two groups, one unmatched and the other matched for age, sex, and PASP)	RVLS of the free wall	45	161 with CPAH (45 matched and 116 unmatched)	Patients with PE had RVLS reduced in all segments of the RV-FW * Those with and without McConnell sign had similar apical RVFWS RVFWS, using a cut-off value of −17.9%, can discriminate patients with PE from those with PAH (sensitivity: 88%, specificity: 63%) RVFWS seemed to abolish its discriminative power when assessed in subjects with normal or mildly reduced FAC
Ramberg et al. (2016) [31]	Retrospective, non-consecutive patients	PE vs. control group (age-matched healthy subjects)	Global and regional RVLS and RV PSS	26 (13 had central and 13 had peripherally located PE)	10	Both RVLS and PSS of the basal and middle part of the RV-FW were reduced compared to the control group * Among patients with central or peripheral PE, no statistically significant differences were observed regarding the RVLS
Dahhan et al. (2016) [32]	Retrospective, non-consecutive patients	Patients with PE followed up for 1 month after the index event	Global and regional RVLS	69; 55 were still alive at 1 month	-	Patients who died in the 1st month had significantly affected RVGLS * RVFWS was also reduced in the non-survivor group (*p* = 0.05), with a significant proportion having >−12.5%
Lee et al. (2019) [33]	Prospective, non-consecutive patients	Non-high-risk PE	RVGLS and RVFWS	144	-	RVGLS and RVFWS were independently associated with in-hospital events ^₴^ * RVFWS ≥ −15.85% could predict in-hospital events ^₴^ (sensitivity: 66.7%, specificity: 66.7%, and NPV: 95.4%) RVGLS ≥ −18.95% could predict in-hospital events ^₴^ (sensitivity: 80%, specificity: 64.3%, and NPV: 96.5%)
Kanar et al. (2019) [34]	Prospective, non-consecutive patients	PE at the time of the index event and at 1-year follow-up	PSS of the RV free wall and Global LV PSS	147	−	Patients who died during follow-up had lower RV-FW PSS, LV global PSS, and a higher RV PSSD index compared to those who survived * Among the survivors, RV-FW PSS, LV global PSS, RV PSSD index, and time difference to PSS between RV-FW and LV lateral wall were all improved at the end of follow-up * Difference in time to PSS between RV-FW and LV lateral wall > 46 msec predicted mortality with a NPV of 93.3%
Trivedi et al. (2020) [35]	Retrospective, non-consecutive patients	Patients with PE (at the time of index event) vs. control group (sex-matched healthy subjects)	RVFWS	84	66	Patients with PE had reduced RVLS in all segments of the RV-FW * Adding RVFWS to traditional TTE indices increases sensitivity and specificity for the diagnosis of PE *
Mazur et al. (2020) [36]	Cross-sectional, non-consecutive patients	Patients diagnosed with PE or RVMI	Global and regional RVLS	53 PE, 23 McConnell sign + (30 patients with RVMI, 16 McConnell sign +)	-	Patients with PE and McConnell sign had lower global RVLS, mainly due to the more altered RVFWS *, while IVS strain did not differ (compared to those without McConnell sign) Patients with McConnell sign had a mean value of AR 1.7× greater than those without it RVFWS and IVS strain showed a strong negative relationship
Li et al. (2022) [37]	Case-control, non-consecutive patients	PE before and after thrombolysis vs. control group (sex- and age-matched healthy subjects)	Global and regional RV PSS	73 39—no PH 34 with PH	40	Before treatment and compared to the control group, irrespective of the presence of PH or not, patients with APE had lower global and regional RV PSS values * After treatment, those with PE but without PH had global and segmental RV PSS values similar to that of the control group After treatment, for those with PE and PH, despite being improved, global and regional RV PSS were still impaired compared to the control group *
Wilinski et al. (2023) [38]	Prospective, cross-sectional, non-consecutive patients	Patients referred for CTPA due to high clinical probability of PE. Comparison was made between those diagnosed vs. those who were not diagnosed with PE, while 30-day all-cause mortality was reported	Global and regional RVLS	88	79 patients without PE	Mid-RVFWS, both indexed to BSA and unindexed, was reduced in patients diagnosed with PE * Unindexed to BSA RVLS of both basal segments was reduced in patients with PE who passed away * Concerning the 30-day all-cause mortality, AUC of all the above indices ranged from 0.60 to 0.70 *
Ballas et al. (2023) [13]	Prospective, consecutive patients	Patients diagnosed with PE	RVFWS	73	-	Patients with conventional echocardiographic signs of PE (such as McConnell sign, 60/60 sign, etc.) had a median RFWS of −11.7, whereas those without had a median RFWS of −19.5 * Patients with sPESI score ≥ 1 had lower RVFWS than those with score 0 * In a small group of patients with non-high-risk PE, RVFWS was increased at day 5 after the index event (in comparison to day 1) *

Abbreviations: AR = apical ratio, calculated as the quotient of apical IVS strain divided by the RVFWS; AUC = area under the curve; BSA = body surface area; CI = confidence interval; COPD = chronic obstructive pulmonary disease; CP = cor pulmonale; CPAH = chronic pulmonary artery hypertension; FAC = fractional area change; FW = free wall; HR = hazard ratio; ICU = intensive care unit; IVS = interventricular septum; LV = left ventricle; NPV = negative predictive value; PASP = pulmonary artery systolic pressure; PE = pulmonary embolism; PH = pulmonary hypertension; PSS = peak systolic strain; PSSD = peak systolic strain dyssynchrony; RV = right ventricle; RVEF = right ventricular ejection fraction; RVFWS = right ventricle free-wall strain; RVGLS = right ventricular global longitudinal strain; RVLS = right ventricle longitudinal strain; RVMI = right ventricular myocardial infarction; RVSP = right ventricular systolic pressure; sPESI = simplified pulmonary embolism severity index; TTE = transthoracic echocardiography. * = For the above comparisons, *p*-value was calculated as <0.05. Ɨ = Primary treatment could be thrombolysis, catheter-based pulmonary embolectomy, and/or anticoagulation therapy (they all received continuous i.v. heparin infusions). ¥ = Adverse outcomes were defined as death, cardiopulmonary resuscitation, and acute PE recurrence. ₴ = In-hospital events were defined as in-hospital PE-related death, need of additive treatments, such as thrombolysis or pulmonary artery thromboembolectomy, and need of inotropes due to unstable vital signs.

The peak systolic strain (PSS) of the free wall of the RV was recorded. At baseline, the mid-segment had the lowest values of the PSS, whereas it showed the greatest improvement after treatment, while it was negatively correlated with TAPSE and fractional area change (FAC) of the RV. No data regarding the hemodynamic status of the patients were provided. The analogy of patients in cardiogenic shock demonstrates that the population of PE was highly selected [22].

### 3.2. Studies Focused on Diagnostic Value of Echocardiographic Strain Analysis in PE

Sugiura et al., in 2009, compared 23 selected patients with massive (*n* = 14) and sub-massive (*n* = 9) acute PE (78% female, mean age: 59 ± 16 years), confirmed with CT scan with 23 age- and sex-matched healthy controls. The authors examined if the global and segmental longitudinal RV peak systolic strain (PSS) differed in the two groups. The PE group had initially reduced global PSS, while after treatment it was improved, except the basal RV lateral wall segment; however, regional strain analysis showed that even after PE treatment, the basal and middle RV lateral wall had lower PSS compared to healthy controls (*p* < 0.05 for all the above comparisons). No clinical outcomes were reported [23].

During the following year, Park et al. reported the results of a retrospective study that enrolled 52 patients with cor pulmonale (CP) in the setting of an emergency department. Twenty-four of the patients were diagnosed with high-risk PE, while the others were diagnosed with severe chronic pulmonary obstructive disease (COPD). The two groups were age- but not gender-matched (fewer female patients included in the chronic CP subgroup). Patients with massive PE had severely affected PSS of the middle RV free wall compared to those with COPD (−2.2 ± 13.8% vs. −20.2 ± 9.2%; *p* < 0.001). The authors reported a cut-off limit of −12.2%, with higher values predicting acute CP with decent accuracy [24].

Takamura et al., in 2011, investigated the impact of acute RV_po_ on global LV function. They enrolled 25 consecutive patients (40% female, mean age: 59 ± 16 years) with massive (*n* = 15) and sub-massive (*n* = 10) PE and compared them to 25 healthy age- and sex-matched subjects. Patients had a TTE upon arrival at the hospital. A second TTE was performed 12 (±8) days later. The authors reported reduced global LV PSS in the three contractile directions (radial, longitudinal, and circumferential) in the patient group vs. the control group (*p* < 0.05 for all). After treatment, global LV PSS in the longitudinal and circumferential axis was improved, while global LV radial PSS remained affected. When assessed segmentally, even though no segments showed improvement in radial PSS on the longitudinal axis, the apical and middle part of the lateral LV wall, as well as the apical inferoseptal wall, restored PSS to percentages similar to the control group. PSS in the circumferential axis was restored to percentages not statistically different to that of the control group. Global peak systolic displacement (PSD) in the radial and longitudinal axis was affected at admission and restored after treatment to units comparable to those of the control group (assessment of PSD in the circumferential direction was not possible). This was one of the first studies using 2DSTE in the setting of acute PE, highlighting the importance of the effect of acute RV_po_ in LV function, due to the interventricular interdependence. However, there were no data provided concerning the clinical outcomes of the enrolled patients [25].

Platz et al., in 2012, published the results of a retrospective study investigating if regional RVLS differed between PE and controls. During a 4-year period, 75 non-consecutive patients (58.7% female, mean age: 54 ± 16 years) with acute PE and 30 normal controls (43.3% male, 50 ± 15 years) were included. The 2DSTE was performed in the first 72 h after PE diagnosis. Regional RVLS was reduced in PE patients in all regions of the lateral RV wall as well as in the middle and basal septum. On the contrary, the strain rate was significantly reduced in all segments of the lateral RV wall but not in the septal regions. Global or regional RVLS was similar in patients with or without elevated troponin values and in patients with or without thrombus in the main pulmonary arteries (detected by CTPA). Thirty-six patients had a positive McConnell sign, and these patients had higher RV/LV ratios. Only apical lateral wall RVLS was significantly reduced in patients with a McConnell sign, in comparison to those without it (−10.08% vs. −13.51%; *p* = 0.042). The authors concluded that regional variations of longitudinal strain and the strain rate exist in patients with PE compared to healthy controls. Moreover, patients with the McConnell sign had lower strain values than patients without it. All-cause mortality was 6.7% at 30 days, but the prognostic role of RVLS was not assessed by dedicated statistical analysis. A major limitation of the study was the absence of data regarding blood pressure at PE diagnosis, thus being unable to determine if patients were or were not in shock [26].

In the following year, Ichikawa et al. published their results regarding the use of 2DSTE in patients with acute and chronic RV_PO_ to assess both RV and LV function and dyssynchrony. They retrospectively enrolled 37 patients with massive (*n* = 19) or sub-massive (*n* = 18) acute PE (65% female, mean age: 61 ± 15 years), 36 patients (15% female, mean age: 55 ± 19 years) with chronic pulmonary arterial hypertension (CPAH), and 33 age-matched healthy controls. Sixteen patients of the CPAH group had chronic thromboembolism pulmonary hypertension (CTEPH). The authors reported that TTE was performed in all patients with acute PE before the initiation of treatment. In patients with CPAH, TTE was performed after an estimated symptomatic period of 52 ± 69 months. In comparison to the control group (−26 ± 4%), global RV longitudinal PSS was reduced in both groups (−14 ± 4 in the PE and −16 ± 5% in the CPAH; *p* < 0.05), and this was mainly attributed to the reduced PSS in the apical and lateral segments. A reduction in the middle and basal RV free-wall segments PSS in the PE group compared to the CPAH group was reported. Global LV longitudinal PSS was reduced only in the PE group compared with the other two groups, even though septal segments were affected in both groups of patients (acute PE and CPAH). The reason was the intact PSS in the apical and lateral LV segments in the CPAH group (in the CPAH group, the apical segment of the lateral LV wall had enhanced PSS, in contrast to the control group; *p* < 0.05), and this might serve as an indicator of the LV lateral wall adaptation to chronic RV_po_ development in CPAH cases, where the septum is mainly affected due to the ventricular interdependence. Furthermore, dyssynchrony indices (including RV PSS dyssynchrony and RV peak systolic displacement dyssynchrony), except for LV radial PSS dyssynchrony, were reduced in both examined groups, whereas LV longitudinal PSS dyssynchrony was even more pronounced in the PE group compared to the CPAH group (87 ± 33 vs. 63 ± 22 msec; *p* < 0.05). No data regarding the clinical outcomes of the subjects were included [27].

Published in 2016, another retrospective study examined if RVLS can differentiate acute from chronic RV overload. They included 45 (69% female, mean age: 64 ± 15 years) non-consecutive and non-high-risk PE patients, compared with 45 patients treated for PAH and matched for age, gender, and pulmonary artery systolic pressure, and an unmatched cohort of 116 patients with PAH. Baseline characteristics of the latter PAH group were not provided. Free-wall RVLS was decreased in PE patients compared with patients in matched and unmatched PAH groups (*p* < 0.001 in both comparisons). Free-wall RVLS proved to be a better index to discriminate patients with PE from patients with PAH (matched group) than the McConnell sign and the pulmonary artery systolic pressure of RV. The sensitivity and specificity of the method was 88% and 63%, respectively. Unfortunately, in patients with a normal or mildly reduced fractional area change (FAC > 35%), the discrimination power of RVLS was lost. PE patients also had lower FAC values than PAH patients, and FAC had a sensitivity of 63% and a specificity of 85% to predict the acuity of PH. Reproducibility of free-wall RVLS, as assessed in randomly selected patients of PE and PAH groups, was better for free-wall RVLS than FAC. The addition of RVLS provided incremental value to traditional echocardiographic indices [30].

Ramberg et al., in 2016, retrospectively evaluated RVLS in 13 (85% female, mean age: 64.0 ± 19.5 years) patients with peripheral PE (based on CTPA), 13 (62% female, 66.0 ± 20.0 years) patients with central PE, and 10 (40% female, 63.0 ± 14.4 years) healthy individuals. The middle and basal RVLS of the free wall were reduced in patients with PE compared to controls and similar in the two PE groups. RVLS in the septal and the apical free wall of the RV did not differ significantly. No data regarding risk in sub-categories of patients with PE were provided, and 42.2% of patients were excluded due to suboptimal images [31].

In a retrospective study conducted by Trivedi et al. in 2020, a total of 84 (61% female, mean age: 67 ± 17 years) non-consecutive patients with PE (who had a TTE performed during their index admission and had sufficient image quality, permitting RV speckle-tracking analysis) were studied. The index PE cohort was created by 233 patients with an in-patient echocardiogram in 2 tertiary centers in Australia. The PE group was compared to 66 sex-matched healthy controls. The authors reported that free-wall RVLS was significantly reduced in the PE group in all segments (basal, middle, and apical), and it stood out as the most valuable index to distinguish those with PE from those without (area under the curve: 0.91). After running a multiple logistic regression model for PE, they concluded that adding free-wall RVLS to the traditional TTE indices can significantly improve both the sensitivity and specificity for diagnosis of PE. No data regarding blood pressure and oxygen saturation on admission were reported, and calculation of the simplified PESI score was precluded [35,39].

Mazur et al., in 2020, investigated the RVLS in patients with RV myocardial infarction (MI) or PE, with and without the McConnell sign. Here, 53 patients (42% female, mean age: 59.0 ± 15.1 years) with high- [15] and intermediate-risk [34] PE and 30 patients (10% female, mean age 61.8 ± 10.9 years) with RVMI were enrolled in their study. TTE was performed within 24 h after stabilization in patients with high-risk PE, the same day with CTPA in patients with intermediate-risk PE, and within 24 h after primary percutaneous coronary intervention after RVMI. Global RVLS, regional RVLS, and the ratio of the apical interventricular segment strain to free-wall RVLS strain was calculated (apical ratio, AR). The McConnell sign was present in a comparable percentage of patients with PE and RVMI. Patients with PE and the McConnell sign had lower global RVLS than those without the McConnell sign due to lower values of free-wall RVLS. Pulmonary artery systolic pressure was correlated with global RVLS and free-wall RVLS but not with interventricular septum RVLS. The authors stated that the pressure overload resulted in lower free-wall RVLS and a compensatory increase of interventricular septum RVLS. The higher the AR, the greater the amplitude of the displacement of the apical RV free wall when the apical interventricular septum contracted (appearance of the McConnell sign). The mean values of AR were greater in PE patients with vs. PE patients without the McConnell sign, indicating severe RV overload. The authors concluded that the McConnell sign was visually identified when AR was ≥1.18 and the amplitude of the RV free-wall apical systolic displacement was >5 mm. The percentage of patients with high-risk PE (28.3%) and the high mean pulmonary artery systolic pressure (58.2 (±16.8) mmHg in patients with negative and 64.5 (±18.2) mmHg in patients with positive McConnell sign) indicated that patients with PE were non-consecutive [36].

Li et al. (2022) prospectively enrolled 73 (44% female, mean age: 55.9 ± 8.8 years) patients with PE (having excluded an unknown proportion of patients with PE due to poor image quality or due to comorbidities, such as lung or kidney disease) and compared them to healthy age- and sex-matched controls. Patients with PE were divided into two groups, those with pulmonary hypertension (PH) on TTE (pulmonary artery systolic pressure ≥ 35 mmHg) and those without PH. All patients received thrombolytic therapy (urokinase 20,000 IU/kg within 2 h) and had a TTE performed within 2 h before thrombolysis and a second TTE on the 14th day after thrombolytic therapy. The indication for reperfusion was not clearly stated, while the authors reported that the included subjects were likely to be hemodynamically stable. The mean RV/LV ratio was 0.88 ± 0.74 in patients without and 1.18 ± 0.43 in those with PH. Global and regional RV longitudinal PSS and time to PSS were measured before and after treatment. Both RV strain parameters in the PE groups were lower than in the control group (*p* < 0.05). The reduction was greater in patients with PE and PH and greater for the free wall in comparison to the septal segments. On the other hand, there were no significant differences in strain parameters after treatment in the PE without PH group vs. the control group (*p* > 0.05), while for the PE with PH group, despite being improved, strain parameters remained lower than those in the control group (*p* < 0.05) [37].

Recently, in 2023, our group published the results of 100 consecutive patients with documented PE who underwent strain analysis of the RV using 2DSTE at most 24 h after diagnosis. Two patients died due to hemodynamic instability before a detailed TTE was performed, eight had a poor echocardiographic window, and seventeen did not have adequate images for strain analysis (off-line). Eventually, 73 (47% female) patients had adequate images for free-wall RVLS calculation (73/98, 74%). Forty-two (58%) were above 80 years old. Using a cut-off value of >−20% to be considered as abnormal, we reported that free-wall RVLS was the most common abnormal echocardiographic index suggestive of RV affection (60%), while abnormal conventional indices were detected in 20% of the patients (*p* < 0.001). There was a significant difference in free-wall RVLS values between patients with a simplified pulmonary embolism severity index (PESI) of 0 and those with a score ≥ 1 (−20.000 (−24.000, −17.800) vs. −17.400 (−21.525, −12.900), respectively; *p* < 0.001). There was a correlation between free-wall RVLS and brain natriuretic peptide levels (BNP). In a small number of non-consecutive patients (*n* = 9) with non-high-risk PE, the 2DSTE study was repeated on days 3 and 5 after the index event. There was a rapid change in free-wall RVLS values, and values on day 5 differed significantly from those on day 1 (*p* < 0.001). However, the lack of a control group (e.g., patients with suspected PE but another final diagnosis) and follow-up data to assess the clinical prognostic consequences of the index were the main limitations of this study [13].

In studies investigating strain analysis in patients with documented PE (for diagnostic or prognostic reasons), the proportion of patients who underwent 2DSTE ranged from 36 to 100%. However, in some studies, the total group of PE subjects and, specifically, the number of patients who were diagnosed with PE were not made clear, and echocardiographic strain analysis was not feasible, and the reasons for that were not clearly stated (Table 2).

### 3.3. Studies Investigated the Prognostic Value of Echocardiographic Strain Analysis in PE

A decade ago, in a prospective study conducted by Vitarelli et al., 66 patients (52% female, mean age: 53 ± 11 years) with intermediate-risk PE (echocardiographic signs of RV dysfunction) were compared with 66 healthy age- and sex-matched controls regarding the RV function. Echocardiography was performed at the onset of the acute episode, after 30 days and 6 months later. Global RVLS, mid-free-wall RVLS, and basal-free-wall RVLS were lower in patients with PE than in controls. Mid-free-wall RVLS showed reversal at 30-day follow-up, earlier than other echocardiographic parameters (RV systolic pressure and RV ejection fraction measured by three-dimensional echocardiography were improved significantly at 6 months). An association between mid-free-wall RVLS and adverse outcomes (defined as death, cardiopulmonary resuscitation, or acute PE recurrence) was observed. Fifteen out of a total eighteen patients who had an adverse event had a mid-free-wall RVLS > −12% (~83% vs. 29% in the group with no adverse events; *p* = 0.002). The combination of mid-free-wall RVLS > −12%, RV systolic pressure > 43 mmHg, and three-dimensional RV ejection fraction < 40% was seen in 94% of patients with adverse events and in 23% of patients without. Moreover, this study was the first that reported an association between RVLS and prognosis in PE. Mid-free-wall RVLS had better accuracy for predicting adverse events compared with global RVLS and conventional indices, such as TAPSE, the RV/LV ratio, or pulmonary acceleration time. However, the control group consisted of healthy individuals and not patients with suspected PE and other final diagnoses [28].

In 2015, Khemasuwan et al. published the results of a retrospective study concerning the value of a variety of echocardiographic indices in predicting all-cause death in patients diagnosed with acute PE and admitted to an intensive care unit (ICU) at some point during their hospitalization. They enrolled a total of 211 patients (51% female, mean age: 61 ± 15 years), and TTE was performed during the first day after the index event. All patients received anticoagulation, while thrombolytic therapy or surgical embolectomy was considered appropriate for 20 patients. RV strain was obtained in 203 patients, but only half of them had optimal echocardiographic windows permitting strain analysis. Based on this small proportion of patients, global RV and RV free-wall strain could not be associated with neither short-term (during hospitalization) nor long-term outcomes but were associated with the need for mechanical ventilation during the ICU stay [29].

During the same year, Dahhan et al. retrospectively analyzed data from 69 patients (48% female) diagnosed with acute PE and a median age of 55 (range: 16–95) years. TTE was performed within the first two days after the PE diagnosis. By the end of the first month after the index event, 14 patients died. Clinical parameters and echocardiography-derived indices were assessed as mortality-predictive models. Traditional TTE parameters (such as TAPSE) failed to show statistically significant differences. Both the RV Tei index and global RVLS were significantly affected in the subgroup of patients who died compared to those who survived (*p* < 0.05 for both, while for RVFWS, a *p*-value of 0.05 was reported). RVLS of the basal part of the RV free wall failed to show statistical significance, despite a remarkable tendency to be more affected in the subgroup of non-survivors. The very high percentage of deaths in the first month (~20%), patients at presentation classified as NYHA IV, and/or those with a calculated PESI score ≥ 86 (class III) means that the population was, once again, highly selected and, therefore, interpretation of the results requires prudence [32].

In a prospective study conducted by Lee et al., published in 2019, it was examined whether free-wall RVLS had any predictive value in patients with non-high-risk PE. Here, 200 patients were initially investigated, and 144 (50% female, mean age: 60.3 ± 14.7 years) were eventually included (56 patients were excluded due to hemodynamic instability, suboptimal echocardiographic images, or another final diagnosis). The primary outcome was defined as the in-hospital events of in-hospital PE-related death, need for additive treatments, such as thrombolysis or embolectomy, or need for inotropes. Both free-wall RVLS and global RVLS were independently associated with in-hospital events. Receiving operator characteristics curve analysis revealed that the area under the curve was 0.754 (95%CI: 0.621–0.887; *p* = 0.001) for free-wall RVLS and 0.731 (95%CI: 0.593–0.868; *p* = 0.003) for global RVLS. The corresponding value for TAPSE was 0.740 (95%CI: 605–0.874; *p* = 0.002). The event rate was 30.5% in patients with free-wall RVLS ≥ −15.85% and 5.7% in those with free-wall RVLS < −15.85% (*p* < 0.001). However, the study did not provide information on whether early (and how early) RVLS can predict adverse outcomes since echocardiography was performed within one week after the diagnosis of PE [33].

Similarly to the previous study, Kanar et al., in 2019, published the results of their prospective study, which enrolled 147 (51% female, mean aged 58.6 ± 15.5 years) patients with documented PE with CTPA. The authors aimed to evaluate RV electromechanical delay (EMD) and mechanical dispersion using 2DSTE and to investigate their association with one-year mortality. Patients with coronary artery disease, atrial fibrillation, LV ejection fraction < 50%, atrio-ventricular conduction disturbances, or poor echocardiographic windows were excluded. ΤΤΕ was performed at the time of the diagnosis and at the end of the follow-up. Forty-four (~30%) patients died before the end of the follow-up period, and investigators reported that those who died had a lower longitudinal free-wall RV and LV peak systolic strain (−13.6 ± 3.6% vs. −18.4 ± 4.6%, *p* < 0.001, and −19.2% ± 4.8% vs. −17.1 ± 4.6%, respectively) and higher RV peak systolic strain dispersion index (35.1 ± 17.5 vs. 68.6 ± 26.0; *p* = 0.001). Between the survivors, free-wall RV PSS resolved at the end of the follow-up (−23.1 ± 4.5 vs. –18.4 ± 4.6 at diagnosis; *p* < 0.001). The difference in time in longitudinal PSS between the free wall of the RV and the lateral wall of the LV was an independent predictor of mortality. The study population consisted of consecutive patients with PE, and mechanical ventilation was performed in 19.1% of the patients, with in-hospital mortality being extremely high (20.4%), while the time that 2DSTE was performed was not precisely stated (“the time of diagnosis” may be before treatment, immediately after treatment, or during hospitalization) [34].

Another cross-sectional observational study, published in 2023 by Wiliński et al., enrolled 167 (55.5% female) selected patients (aged 69.5 ± 15.3 years) with suspected PE (high clinical probability) who were referred for CTPA. PE was diagnosed in 88 patients (5/88 cases were massive PE). All patients underwent a TTE within 24 h of admission. The clinical endpoint was death from any cause in the first 30 days. During the follow-up period, 12 PE patients died, and in 5, their death was attributed to either RV failure due to APE or as a complication of thrombolysis administered for the PE. Patients with PE had lower values of RVLS of the mid-segment of the free wall (both indexed to the body surface area (BSA) and unindexed) compared to subjects without PE (*p* = 0.03 and 0.01, respectively). Patients diagnosed with PE who passed away had lower values of RVLS at the basal segments of both the free wall and interventricular septum (*p* = 0.03 and 0.049, respectively). Echocardiographic strain predictors of 30-day mortality included a RVLS of the middle segment of the free wall > −21% or > −14% when indexed to the BSA (AUC = 0.6 and 0.62, *p* = 0.01 and 0.003, respectively) and non-indexed RVLS of the basal segments of the RV free wall and interventricular septum > −14% and > −15% (AUC = 0.7 and 0.68, respectively, both with *p* = 0.02). Among several clinical, biochemical, and echocardiographic parameters, the parameter with the best prediction value was the PESI score (area under the curve 0.88; *p* < 0.001). The authors commented that the lower discrimination ability of the RVLS in their study compared with previous studies was possibly because the controls were subjects with suspected PE and not healthy people (of note, almost 40% of the patients that were not diagnosed with PE had chronic heart failure). As stated above, these results cannot be applied in patients with a low or intermediate clinical probability of PE [38].

## 4. Experimental Studies of PE and RV Longitudinal Strain

Interestingly, several experimental models of PE have been developed (e.g., by injecting organized thrombus or microspheres into the venous circulation) [40]. However, only limited data exist regarding the value of RVLS in these models. Morita et al., in 2022, investigated if echocardiographic indices correlated with right heart catheterization values in an experimental model of PE in six dogs. PE was induced by injecting microspheres into the vein circulation. After two days of microsphere injection, the mean pulmonary artery pressure remained elevated, and blood pressure recovered. The RV Tei index remained abnormal and correlated with pulmonary vascular resistance. Fifteen minutes after PE “induction”, free-wall RVLS and global RVLS were reduced. Two days later, RVLS returned to baseline values. Septal RVLS did not change significantly between time 0 (before PE induction) and day 2 [41]. A few years before, the same group had shown that RV 2DSTE-related indices were highly repeatable in dogs [42].

## 5. Interpretation of the Data

**Figure 2:**
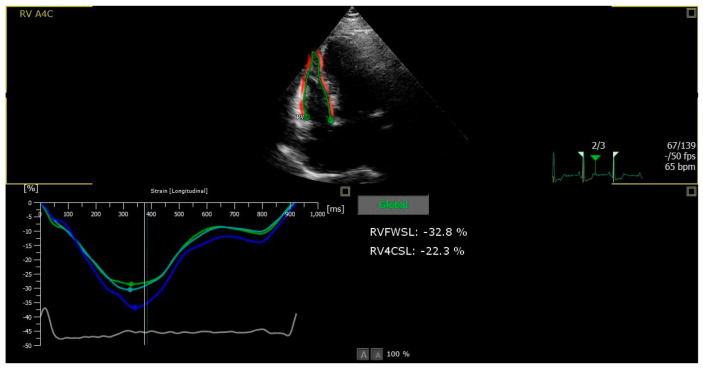
Example of time–strain curves in the RV longitudinal direction in the three free-wall myocardial segments.

Indices of mechanical strain measured by 2DSTE are sensitive for assessment of systolic performance of both ventricles. Due to anatomical and functional characteristics of the RV, indices of RVLS have been used for evaluation of RV systolic function and changes after PE. However, measuring only one index of RVLS and using a cut-off value for diagnostic or prognostic reasons in patients with suspected or documented PE is promising but still challenging. Besides, a unanimous deformation-related index would only be a rough approximation for the assessment of right or left ventricle function in any clinical setting. There are more things to consider in RV deformation apart from RVLS. Since (in terms of echocardiography) RV can be divided into six segments, three for the free wall and three for the interventricular septum (that are mostly part of the LV), different aspects of RV deformation can be measured. That means that apart from global RVLS, which is the mean value of peak systolic strain for each of the six RV segments, other segmental strains can be measured, such as RV free-wall longitudinal strain (RVFWLS), which is the mean of peak systolic strain only for the three free-wall segments (Figure 2).

Things may become even more complicated, since (1) strain can be individually measured for every distinct RV segment, (2) in the newest versions of strain-dedicated software (vendor dependent), three-layered strain (endo-, mid-, and epicardial) can also be measured, (3) the advent of real-time three-dimensional echocardiography also allows measurements of RV strain indices, in a faster and easier way through dedicated software (even though the final images are a product of summation and optimization of 2D images), and (4) the constantly increasing use of cardiac magnetic resonance (CMR) has led to CMR-derived strain parameters by means of certain “myocardial tagging” techniques, thus contributing to the overall heterogeneity of methods used [43]. Currently, in real clinical practice, the method has two main limitations when used in such a group of patients, as follows: (a) the poor acoustic window in patients with PE when they are in distress (technical and anatomical reasons), especially in the context of an affected RV, and (b) the fact that RV strain-related indices are not readily available for interpretation and implementation of the results in the clinical setting but need off-line analysis to be measured, thus contributing more to our understanding of the underlying pathophysiology of PE.

**Figure 3:**
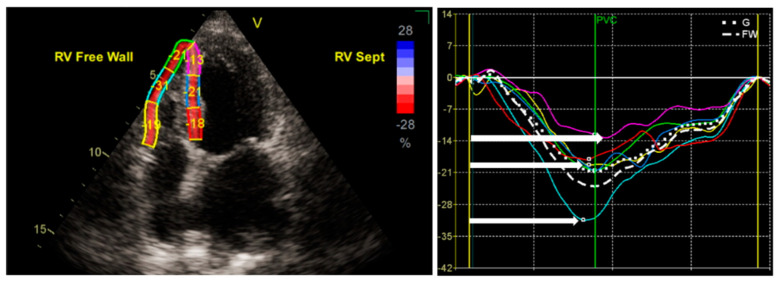
Examples of global (right panel, dotted line) and free-wall RV (right panel, dashed line) longitudinal strain curves. A six-segment model was created (three segments for the free wall and three for the ventricular septum) using a tracking algorithm software after delineation of the endocardial borders in a RV-focused, four-chamber view. Each myocardial segment has a unique color code. White right arrows represent the time to PSS.

What is of most importance is that even though the term “longitudinal strain” is usually conceptually connected to the peak systolic strain of every RV myocardial segment (or the mean value of them), the time of achieving this value (related to timing of pulmonary valve closure) is also important. In terms of pathophysiology, even when a distinct myocardial segment reaches its peak systolic strain value, the timing is important, since every delay is translated to the myocardial segmental systole against a closed pulmonic valve. This can be measured in two ways: (1) net time after pulmonary valve closure, and (2) net time after a standard time point (which usually is the peak of the R wave in the electrocardiogram; Figure 3).

Keeping all this in mind, not any single strain-related index can be used on its own in the context of different clinical scenarios of PE. A combination of indices might be more useful in clinical practice (e.g., indices considering both RVLS as an indicator of RV deformation and troponin as an indicator of RV injury).

## 6. Conclusions

Echocardiography and its “traditional” RV_d/PO_ indices are useful to evaluate the cause of shock in high-risk patients with suspected high-risk PE. It is also a risk modifier in patients who receive a diagnosis of PE. Strain of the RV free wall and, more specifically, strain of the middle part of the RV free wall, either expressed as RVLS or as PSS, has been shown—through several studies—to be correlated with either mortality and/or as an aid tool to differentiate PE from other diagnoses. Recovery of an initially affected strain of the RV free wall was also observed after treatment administration. Furthermore, strain of the RV free wall was also reported to correlate with other commonly used prognostic indices, such as the PESI score and BNP. Most of the authors stated that strain of the RV—as a method—showed decent intra- and inter-observer variability, while also being angle-dependent, making repeatability of the measurements feasible. Unfortunately, a variety of limitations exist for all of the included studies. The necessity of better-structured, multi-center studies consisting of larger representative populations of all PE risk categories and comparing them to age- and sex-matched healthy—and more realistic—populations with comorbidities is mandatory.

## Figures and Tables

**Table 2:** A summary of the proportion of patients diagnosed with APE (per study and in total) who successfully had RV free-wall strain measured (both RVLS and PSS are included; studies are sorted by the year they were published, from older to the most recent).

First Author (Published Year) [Ref.]	Type of Strain Used	Total (Referred) Number of Patients with APE Evaluated *	Total Number of Patients with APE in Whom RV Free-Wall Strain Calculation Was Obtained ^∆^	Proportion of Patients (%)
Kjaergaard et al. (2004) [21]	RVLS of the RV free wall	3	3	100
Park et al. (2007) [22]	PSS of the RV free wall	28	24	85.7
Sugiura et al. (2009) [23]	Global and regional RV PSS	24	23	95.8
Park et al. (2010) [24]	Regional RV PSS	24	24	100
Platz et al. (2012) [26]	Global and regional RVLS	84	75	89.3
Ichikawa et al. (2013) [27]	Global and regional RV and LV PSS	40	37	92.5
Vitarelli et al. (2014) [28]	RVGLS and RVFWS	76	66	86.8
Khemasuwan et al. (2015) [29]	Global and regional RVLS	235	110	46.8
Wright et al. (2016) [30]	RVLS of the free wall	45 (Not stated clearly)	45	100
Ramberg et al. (2016) [31]	Global and regional RVLS and RV PSS	45	26	57.8
Dahhan et al. (2016) [32]	Global and regional RVLS	35	69	51.1
Lee et al. (2019) [33]	RVGLS and RVFWS	170	144	84.7
Kanar et al. (2019) [34]	PSS of the RV free wall and Global LV PSS	147 (Not stated clearly)	147	100
Trivedi et al. (2020) [35]	RVFWS	233	84	36
Mazur et al. (2020) [36]	Global and regional RVLS	53 (Not stated clearly)	53	100
Li et al. (2022) [37]	Global and regional RV PSS	73 (Not stated clearly)	73	100
Wilinski et al. (2023) [38]	Global and regional RVLS	194	167	86.1
Ballas et al. (2023) [13]	RVFWS	100	73	73
**Summary of the studies**		1709	1243	72.7
**Summary of the studies having excluded those where the initial PE population was not clearly stated**		1391	925	66.5

Abbreviations: APE = acute pulmonary embolism; LV = left ventricle; PSS = peak systolic strain; RV = right ventricle; RVFWS = right ventricle free-wall strain; RVGLS = right ventricular global longitudinal strain; RVLS = right ventricle longitudinal strain. * = We have included the total of patients diagnosed with PE who were evaluated in each study. Out of all those, there were many in whom RV free-wall strain was not assessed due to the fact that they were excluded by the authors for different reasons each time, and except for having a suboptimal or poor echocardiographic window for strain acquisition (there were times where the exclusion criteria were not clearly reported). ^∆^ = Some of the authors reported data concerning RV strain-derived indices both at the time of the index event and during the follow-up, where some of the subjects may have died before the new echocardiogram was performed. That being said, we have included here all the subjects in whom RV free-wall strain was obtained at least one time and the strain-derived values were taken into account in the reported data analysis.
